# Seroprevalence of Zika Virus in Amphawa District, Thailand, after the 2016 Pandemic

**DOI:** 10.3390/v14030476

**Published:** 2022-02-25

**Authors:** Salin Sirinam, Supawat Chatchen, Watcharee Arunsodsai, Suriya Guharat, Kriengsak Limkittikul

**Affiliations:** 1 Department of Tropical Pediatrics, Faculty of Tropical Medicine, Mahidol University, Bangkok 10400, Thailand; salin.sir@mahidol.ac.th (S.S.); supawat.cht@mahidol.ac.th (S.C.); watcharee.cho@mahidol.ac.th (W.A.); 2 Ministry of Public Health, Nonthaburi 11000, Thailand; suriyanaranong@gmail.com

**Keywords:** Zika virus, flavivirus, epidemiology, ELISA, PRNT, Thailand

## Abstract

In 2016, Zika virus (ZIKV) infection was declared a public health emergency of international concern because of the neurological consequences in babies born to infected people. Because of the mild and nonspecific symptoms, serological tests are essential in epidemiological studies. However, cross-reactive antibodies between other Flaviviridae members may complicate the interpretation of results of these tests. This study investigated the seroprevalence of ZIKV infection in Samut Songkhram in central Thailand which was affected by the Zika outbreak of 2016. Three hundred and fifty volunteers aged 5–50 years in Amphawa District, Samut Songkhram, were enrolled between April 2017 and April 2018. ZIKV nonstructural protein 1 (NS1) immunoglobulin G enzyme-linked immunosorbent assay (ELISA) was used to screen serum samples collected on the first day of enrollment and after 6 and 12 months. The seroprevalence and seroconversion of ZIKV were assessed. Cases of ZIKV seroconversion were verified as evidence of ZIKV infection by NS1 blockade-of-binding ELISA and plaque reduction neutralization test (PRNT50). ZIKV seroprevalence in Amphawa was 15.1–17.8% with no significant change over the year. The total seroconversion rate throughout the year was 7/100 person-years. The ratio of asymptomatic to symptomatic infections was 4.5:1. The cases in our study confirmed the occurrence of occult ZIKV infections in the community. These undetected infections might promote the spread of ZIKV in vulnerable groups of the community.

## 1. Introduction

Zika virus (ZIKV) is a mosquito-borne flavivirus in the family Flaviviridae. It was first isolated from a rhesus monkey in the Zika Forest, Uganda, in 1947 [1]. The first human case was also detected in Uganda between 1962 and 1963 [2]. The virus migrated to Southeast Asia and was first discovered in mosquitoes in 1966 [3]. In 2007, a ZIKV cluster was reported in the Yap Islands, the Federated States of Micronesia, followed by an outbreak in the Pacific region in 2013–2015 [4,5]. After spreading to Brazil in 2015, the impact on public health was recognized when the neurological consequences were found in neonates born to infected mothers [6,7]. As a result, the World Health Organization declared ZIKV infection a public health emergency of international concern in February 2016 [8].

The majority of ZIKV infections are asymptomatic or mild and self-limiting. Common symptoms include rash, arthralgia, mild fever, conjunctivitis, headache, and myalgia, which are similar to those of *Aedes* mosquito-borne viral infections, e.g., dengue fever [4,9,10]. Thus, clinical assessment alone is insufficient to diagnose ZIKV infection, posing a challenge to the surveillance system. Moreover, the interpretation of serological results is complicated because of *Flavivirus* cross-reactivity [11,12]. Enzyme-linked immunosorbent assay (ELISA) targeting nonstructural protein 1 (NS1) in the ZIKV immunoglobulin (IgG) has displayed potential as a serological test for diagnosing ZIKV infection [13,14]. As is the case in many regions, the incidence rate of ZIKV infection has remained unclear in Thailand. The finding of acute ZIKV infection among residents from different regions in Thailand in 2012–2014 supported the endemic transmission throughout the country [15]. In fact, ZIKV has been circulating at a low but consistent level in Thailand since at least 2002, according to molecular epidemiological and genetic diversity studies in mosquitoes and patients [16,17].

The 2016 ZIKV pandemic resulted in approximately 2300 confirmed cases in 43 provinces in Thailand in 2016–2018. Samut Songkhram, located in central Thailand, was one of the provinces affected by Zika [18]. However, most of the reported cases were identified via passive surveillance, tracing from the people who recognized their symptoms. Thus, the reported number of Zika cases could be underestimated. We, therefore, conducted a prospective study to explore the seroprevalence in this area after the Zika disease pandemic using NS1 IgG ELISA.

## 2. Materials and Methods

### 2.1. Study Site and Sample Collection

**Figure 1:**
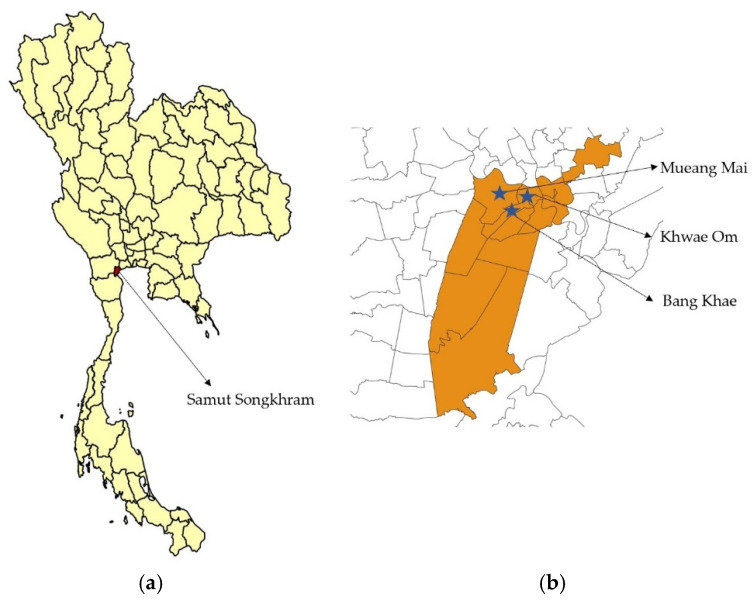
(**a**) The location of Samut Songkhram Province, Thailand. (**b**) The map of Amphawa District. The blue stars mark three subdistricts from which the participants were recruited.

A cohort study was conducted in the population aged 5–50 years in Amphawa District, Samut Songkhram Province (Figure 1). Patients with any immunosuppressive condition or history of blood component transfusion within 3 months before enrollment were excluded. The study was approved by the Ethics Committee of the Faculty of Tropical Medicine, Mahidol University, under protocol TMEC 16-107 and the Human Research Ethics Committee of Samut Songkhram Health Office, Ministry of Public Health, under protocol 1/2560.

**Figure 2:**
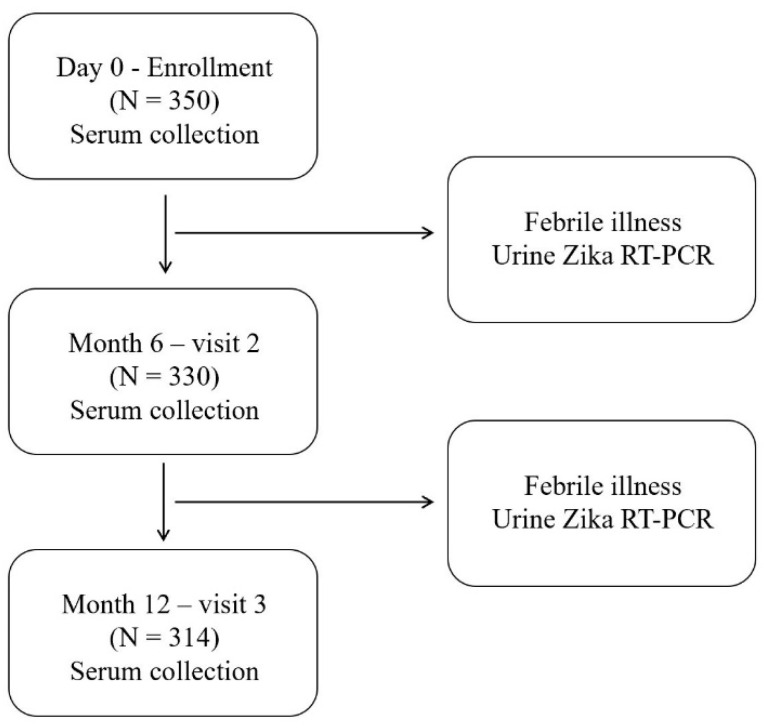
Schematic diagram of the study design and procedures. Zika virus (ZIKV) and dengue virus nonstructural protein 1 antibodies were examined at enrollment (Day 0) and after 6 and 12 months. Urine ZIKV RT-PCR was performed for volunteers who visited a hospital with acute febrile illness.

We finally recruited 350 volunteers into the study (Figure 2). The history of previous ZIKV, dengue virus, and Japanese encephalitis virus infection; any febrile illness; and yellow fever, dengue, and Japanese encephalitis vaccination was reviewed. We collected a baseline blood sample on the first day of enrollment and during follow-up visits at 6 and 12 months (from April 2017 to April 2018) for ELISA to detect ZIKV NS1 and dengue virus NS1.

Additionally, passive febrile surveillance was performed during the 12 months of follow-up. Individuals who experienced acute febrile illness (oral temperature ≥ 38 °C for more than 48 h without localizing symptoms) were assessed via reverse transcription–polymerase chain reaction (RT-PCR) using urine to identify ZIKV infection.

All the serum and urine samples were tested and stored at −80 °C at the Department of Tropical Pediatrics, Faculty of Tropical Medicine, Mahidol University, until analysis.

### 2.2. ZIKV and Dengue Virus NS1 IgG ELISA

ELISA was performed to detect ZIKV and dengue virus NS1 IgG using serum samples (Day 0, Month 6, and Month 12) to evaluate the immune status of the participants. Briefly, 96-well ELISA plates (Corning Life Sciences, Corning, NY, USA) were filled with 60 µL/well of ZIKV or dengue NS1 proteins (500 ng) in 0.018 M carbonate buffer. The subsequent processes were described previously [14]. Finally, the optical density (OD) of the wells at 450 nm was measured using an ELISA plate reader.

### 2.3. ZIKV NS1 Blockade-of-Binding (BOB) ELISA

ZIKV NS1 BOB ELISA measures the levels of serum antibodies that block the binding of a highly specific mAb to ZINV NS1 as described previously [19]. Briefly, 96-well flat-bottom microtiter plates (Corning Life Sciences, Corning, NY, USA) were coated with ZIKV NS1 in a carbonate/bicarbonate buffer (pH 9.6 ± 0.1) overnight at 4 °C. The plates were blocked with PBS-T supplemented with 1% (*v*/*v*) BSA for 60 ± 5 min at 25 °C. A solution containing 50 µL of serum (1:10 dilution) or the ZIKV NS1-specific antibody ZKA35 (Ab1036-10.0) (Absolute Antibody, Oxford, UK) as a positive control at 5 μg/mL prepared in PBS was immediately mixed. The plates were incubated for 60 ± 5 min at 25 °C with peroxidase-conjugated ZKA35-HRP prepared in PBS. The plates were washed with PBS-T and developed with SureBlue^™^ TMP (KPL Inc., Gaithersburg, MD, USA) for 20 ± 10 min at 25 °C. The reaction was stopped by adding 0.2 M sulfuric acid (Thermo Fisher Scientific, Fair Lawn, NJ, USA), and the plates were read using a microplate reader at 450 nm. For the assay result, the percentage of blockade inhibition was calculated using the following equation: ((ODsample − ODnegative control)/(ODpositive control − ODnegative control)) × 100.

### 2.4. Plaque Reduction Neutralization Test (PRNT)

The serum samples were tested for specific antibodies against dengue virus serotypes 1–4 (DEN-1 strain 16007, DEN-2 strain 16681, DEN-3 strain 16562, DEN-4 strain C0036/06) and ZIKV (strain SV0127/14) in LLC-MK2 cells using the 50% plaque reduction criterion as described previously [20]. Briefly, a monolayer of LLC-MK2 cells was cultivated in 12-well plates (Corning Life Sciences, Corning, NY, USA). Serum samples were inactivated (56 °C for 30 min) and serially diluted to 1:10, 1:40, 1:160, 1:640, and 1:2560. Each ZIKV and dengue virus serotype was then separately added into diluted serum, and serum–virus mixtures were incubated at 35 °C for 60 min. The mixtures were then inoculated onto monolayer LLC-MK2 cells in 12-well plates. After 4 days of incubation, neutral red was used to stain the inoculated cells. Each serum sample was tested in duplicate, and the number of plaque-forming units was recorded as the average of two cultures. PRNT50 was calculated using the probit model using SPSS version 18.0 (SPSS, Inc., Chicago, IL, USA). The PRNT50 endpoint titers were expressed as the reciprocal of the last serum dilution.

### 2.5. ZIKV RT-PCR

For ZIKV RT-PCR, urine samples from the participants with acute febrile illness were selected for RNA extraction using a QIAGEN Viral RNA Kit (Qiagen, Hilden, Germany) according to the manufacturer’s instructions. The processes were performed as previously described [21].

### 2.6. Criteria of ZIKV NS1 Seropositivity and Seroconversion

P/N ratio was defined as the OD of the sample to the OD of the negative control.

The positive ZIKV NS1 antibody was defined as the ratio of the OD of the sample to the OD of the negative control > 2 (Zika NS1 P/N ratio > 2) and the ratio of the ZIKV NS1 P/N ratio to the dengue NS1 P/N ratio > 2 (ZIKV/dengue NS1 P/N ratio > 2).

The negative ZIKV NS1 antibody was defined as either the ratio of the OD of the sample to the OD of the negative control < 2 (Zika NS1 P/N ratio < 2) or the ratio of the ZIKV NS1 P/N ratio to the dengue NS1 P/N ratio < 2 (ZIKV/dengue NS1 P/N ratio < 2).

ZIKV NS1 seroconversion described a change from negativity to positivity for the ZIKV NS1 antibody. Definite seroconversion was characterized by a greater than twofold increase of the ZIKV NS1 P/N ratio in two consecutive serums, whereas a < twofold increase in the ratio was defined as borderline seroconversion.

### 2.7. Statistical Analysis

The analysis was performed using SPSS version 18.0 (SPSS Inc., Chicago, IL, USA). The Mann–Whitney U test was used to assess differences between the groups; *p* value < 0.05 was considered statistically significant.

## 3. Results

### 3.1. The Seroprevalence of ZIKV Infection

The population was recruited from three subdistricts of Amphawa, Samut Songkhram Province, surrounding the outbreak area. The demographic data of the participants on the first day of enrollment (Day 0) are presented in Table 1. In total, 20 and 36 participants (5.7% and 10.3%, respectively) missed the follow-up visits at 6 and 12 months, respectively.

Of the 350 participants, 15.1% were positive for the ZIKV NS1 antibody on Day 0. Testing of the blood samples collected after 6 and 12 months demonstrated that the prevalence of the Zika NS1 positive antibody increased to 17.2% and 17.8%, respectively. No significant changes in the seroprevalence were observed between the two consecutive visits. An increasing seroprevalence of ZIKV NS1 was also observed among the age groups of 16–30 years and 31–50 years (Table 2).

### 3.2. The Seroconversion Rate of the ZIKV NS1 Antibody

**Figure 3:**
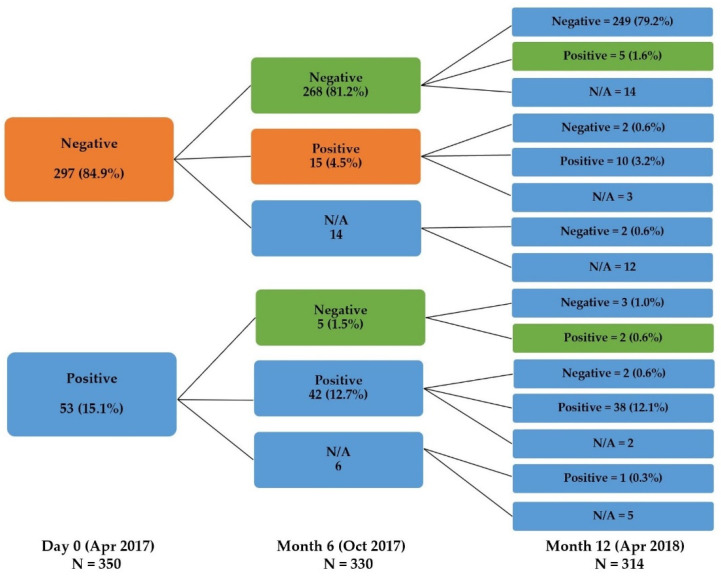
The change in the serostatus of the participants at each visit. The orange color represents the proportion of seroconverted participants between Day 0 and Month 6. The green color represents subjects with seroconversion between Month 6 and Month 12 (N/A: participants lost to follow-up).

We further explored the seroconversion rate in this cohort using the criteria described in the Methods section. Fifteen of the 330 participants (4.5%) had seroconversion during the first 6 months, whereas 7 of the 314 participants (2.2%) exhibited seroconversion during the next 6 months (Figure 3).

Definite seroconversion was identified in 12 participants. The relative inhibition of the ZIKV NS1 BOB ELISA and ZIKV PRNT50 titers was comparatively consistent (Table 3). However, all the participants exhibited PRNT50 positivity for dengue virus at enrollment.

Meanwhile, 18 of the 22 seroconverted participants denied any ZIKV-suspected illness during the study. The other four subjects (ID codes 173, 191, 210, 236) retrospectively reported acute febrile illness or rash with or without red eyes that had occurred before the follow-up visits. All of these patients displayed definite seroconversion (Supplementary Table S1).

To investigate the characteristics of the participants with definite ZIKV NS1 seroconversion, a seroconversion map was generated (Supplementary Figure S1). We found one cluster including two symptomatic (ID codes 173 and 191) and five asymptomatic (ID codes 166, 167, 168, 192, 196) infections that developed between Day 0 and Month 6 of the study.

Concerning the passive febrile surveillance, 72 participants reported 101 febrile illness episodes during the study. Eight of these episodes (7.9%) required hospital visits, but no patients exhibited positivity for ZIKV in urine by RT-PCR.

## 4. Discussion

Evidence of ZIKV transmission in Thailand was reported before the 2016 pandemic [15]. We performed this prospective study to examine the seroprevalence and seroconversion rate after the 2016 Zika outbreak in Amphawa District, Samut Songkhram. No cases of Zika were reported in this area during our investigation; however, the incidence could be underestimated because of the mild clinical manifestation of the disease in many cases. An epidemiological survey could provide helpful information to evaluate the burden of infection in a certain area. In fact, because of the high background of dengue virus seropositivity, interpreting ZIKV immunity in an endemic area is more complex. We developed ZIKV NS1 ELISA and achieved comparable results with the globally available NS1 BOB ELISA, including minimal cross-reactivity with other flaviviruses. Using our ZIKV NS1 ELISA and cutoff criteria, the seroprevalence after the outbreak (i.e., Day 0) was much lower than that reported in other studies [22,23,24,25,26]. We also observed an increase in ZIKV NS1 seroprevalence throughout the age groups, which was consistent with prior findings [27]. Nevertheless, the seroprevalence in our cohort did not significantly change during the 12-month study period. By contrast, Handerson et al. found that the seroprevalence of ZIKV in French Polynesia and Fiji significantly declined over 18 months in adults but persisted in children [28]. Although we do not have sufficient information on the seroprevalence of Zika in Thailand prior to the 2016 outbreak, it is unclear whether the absence of changes in seroprevalence in this study were related to antibody persistence or a natural boost of immunity in this area. This raised the awareness of using a serological test to probe the history of ZIKV infection in a suspected individual.

Furthermore, the seroconversion rate in this study appeared to be higher in April–October, which is the rainy season in Thailand. This indicates that the peak transmission is related to the season. Considering that 4 of the 22 seroconverted participants reported acute febrile illness and rash, the ratio of asymptomatic to symptomatic infections was 4.5:1. This proportion was similar to that reported previously by Duffy et al. who estimated that the ratio was 4:1 during the 2007 outbreak in Yap Islands [4]. However, our data are limited by the fact the seroconverted subjects reported their illness retrospectively, and they did not have their serum or urine samples confirmed for ZIKV infection by RT-PCR. Thus, the ratio in our cohort could represent over- or underestimation. The Department of Disease Control of the Ministry of Public Health of Thailand reported that the number of asymptomatic ZIKV infections was much lower than that of symptomatic infections in 2016–2018 [18]. This implies that a large number of individuals with undetected asymptomatic infection could be present in the community. They could transmit the virus to more vulnerable persons nearby, such as pregnant women and babies. One study found that babies born to asymptomatic Zika-infected mothers could experience neurological consequences [29]. Hence, active surveillance in areas of outbreaks is crucial.

The ZIKV PRNT titers in our cohort were consistent with the ZIKV NS1 antibody titers and NS1 BOB data. However, the interpretation was difficult because of high cross-reactivity between ZIKV and other dengue virus serotypes. Recent studies in Thailand also demonstrated cross-reactivity between neutralizing antibodies against ZIKV and all four dengue virus serotypes [25,26]. Therefore, PRNT may not be an appropriate tool to confirm ZIKV infection in dengue-endemic areas.

The seroconversion map demonstrated that the cluster of individuals with ZIKV infection concentrated in one neighborhood. This information supports the plan of active surveillance in the outbreak region and further investigation to assess the full scope of disease transmission.

Our study confirmed the wide range of disease severity. Infected individuals may be asymptomatic or decline to seek medical services if they only have mild symptoms. However, they can spread the infection to vulnerable groups in the community. For a better understanding of Zika in the future, more research on the serostatus of ZIKV infection in Thailand as well as on the dynamics, nature, and duration of immunity protection against ZIKV is needed.

## Figures and Tables

**Table 1:** The demographic characteristics of the study subjects.

Subdistrict	Number	Sex		Age (Years)		
		Male	Female	5–15	16–30	31–50
Mueang Mai	124	53 (42.7%)	71 (57.3%)	20 (16.1%)	36 (29.0%)	68 (54.9%)
Khwae Om *	105	45 (42.9%)	60 (57.1%)	28 (26.7%)	27 (25.7%)	50 (47.6%)
Bang Khae	121	45 (37.2%)	76 (62.8%)	24 (19.8%)	34 (28.1%)	63 (52.1%)
Total (% **)	350	143 (40.9%)	207 (59.1%)	72 (20.6%)	97 (27.7%)	181 (51.7%)

* One participant from Khwae Om subdistrict had a history of RT-PCR-confirmed Zika virus infection 6 months before recruitment. ** The percentages were the proportions of each characteristic category in the column and the total number of the subjects at the enrollment (350).

**Table 2:** The number (%) of subjects who had Zika virus nonstructural protein 1 seropositivity by age group and visit.

Age (Years)	Day 0 (N = 350)	Month 6 (N = 330)	Month 12 (N = 314)	*p*	
				Day 0 vs. Month 6	Month 6 vs. Month 12
5–15	8/73 (11.0%)	11/71 (15.4%)	7/64 (10.9%)	0.577	0.600
16–30	13/97 (13.4%)	16/88 (18.1%)	17/83 (20.5%)	0.490	0.852
31–50	32/180 (17.8%)	30/171 (17.5%)	32/167 (19.2%)	1.000	0.807
Total	53 (15.1%)	57 (17.2%)	56 (17.8%)	0.516	0.933

**Table 3:** The results of the Zika virus nonstructural protein 1 (NS1) blockade-of-binding (BOB) ELISA and the neutralizing antibodies against Zika virus and dengue virus serotypes detected by PRNT50 among the 12 participants with definite seroconversion.

ID Code	Laboratory Testing		Day 0 (April 2017)	Month 6 (October 2017)	Month 12 (April 2018)
95	% inhibition	Zika NS1-BOB	26.0	98.3	72.7
	PRNT50	Zika	198	>2560	>2560
		DEN1	29	>2560	295
		DEN2	259	>2560	382
		DEN3	241	>2560	489
		DEN4	196	>2560	311
166	% inhibition	Zika NS1-BOB	19.6	92.6	96.9
	PRNT50	Zika	16	>2560	280
		DEN1	1843	>2560	>2560
		DEN2	1780	>2560	>2560
		DEN3	>2560	>2560	>2560
		DEN4	82	133	591
		DEN4	1711	1440	5
167	% inhibition	Zika NS1-BOB	1.5	82.5	67.6
	PRNT50	Zika	<10	1307	572
		DEN1	10	1405	192
		DEN2	1080	>2560	863
		DEN3	107	2096	892
		DEN4	<10	762	114
		DEN4	114	320	110
168	% inhibition	Zika NS1-BOB	12.5	80.3	48.3
	PRNT50	Zika	<10	1637	259
		DEN1	>2560	22	432
		DEN2	>2560	209	1459
		DEN3	754	60	753
		DEN4	1014	52	250
192	% inhibition	Zika NS1-BOB	36.7	84.6	71.2
	PRNT50	Zika	<10	1298	1541
		DEN1	782	>2560	1929
		DEN2	535	>2560	>2560
		DEN3	1076	>2560	1298
		DEN4	29	19	31
196	% inhibition	Zika NS1-BOB	40.3	94.5	85.9
	PRNT50	Zika	<10	2083	949
		DEN1	<10	316	174
		DEN2	66	>2560	1022
		DEN3	1298	474	560
		DEN4	<10	74	48
232	% inhibition	Zika NS1-BOB	15.7	82.1	81.8
	PRNT50	Zika	207	>2560	2439
		DEN1	262	2157	1406
		DEN2	276	2062	1315
		DEN3	39	905	1435
		DEN4	132	1570	2504
173	% inhibition	Zika NS1-BOB	20.3	70.0	39.3
	PRNT50	Zika	<10	>2560	745
		DEN1	592	>2560	1311
		DEN2	966	>2560	1353
		DEN3	334	>2560	1075
		DEN4	60	1128	166
191	% inhibition	Zika NS1-BOB	46.3	103.8	97.1
	PRNT50	Zika	1520	>2560	1628
		DEN1	307	>2560	2461
		DEN2	2217	>2560	>2560
		DEN3	465	>2560	>2560
		DEN4	277	2527	536
210	% inhibition	Zika NS1-BOB	49.4	106.0	missing
	PRNT50	Zika	210	>2560	missing
		DEN1	<10	830	missing
		DEN2	<10	2458	missing
		DEN3	<10	917	missing
		DEN4	>2560	520	missing
236	% inhibition	Zika NS1-BOB	28.0	53.9	38.8
	PRNT50	Zika	<10	1103	>2560
		DEN1	<10	47	17
		DEN2	<10	88	540
		DEN3	<10	55	424
		DEN4	37	56	169
139	% inhibition	Zika NS1-BOB	28.4	20.6	60.4
	PRNT50	Zika	106	752	1641
		DEN1	55	63	1428
		DEN2	622	475	>2560
		DEN3	249	121	760
		DEN4	121	111	570

## Supplementary Materials

The following supporting information can be downloaded at https://www.mdpi.com/article/10.3390/v14030476/s1, Table S1: Zika virus and dengue virus nonstructural protein 1 (NS1) OD of the sample/OD of the negative control ratio (P/N ratio) of 22 Zika seroconverted participants, Figure S1: The seroconversion map presents the distance and areas in which the participants with definite Zika seroconversion were living in the community. The changes in the participants’ serostatus that occurred within 6 months are presented in red. One participant with definite seroconversion between the 6- and 12-month visits is highlighted in blue. The map demonstrates a cluster of two symptomatic (ID codes 173 and 191) and five asymptomatic (ID codes 166, 167, 168, 192, 196) infections.
