# Supplementary material for: Seroprevalence of Zika Virus in Amphawa District, Thailand, after the 2016 Pandemic
Source: Viruses. 2022 Feb 25;14(3):476. doi: 10.3390/v14030476 (PMC8953292; doi:10.3390/v14030476)
Supplement: Supplementary file 1 [file viruses-14-00476-s001.zip › viruses-1586007-figure S1.pdf]

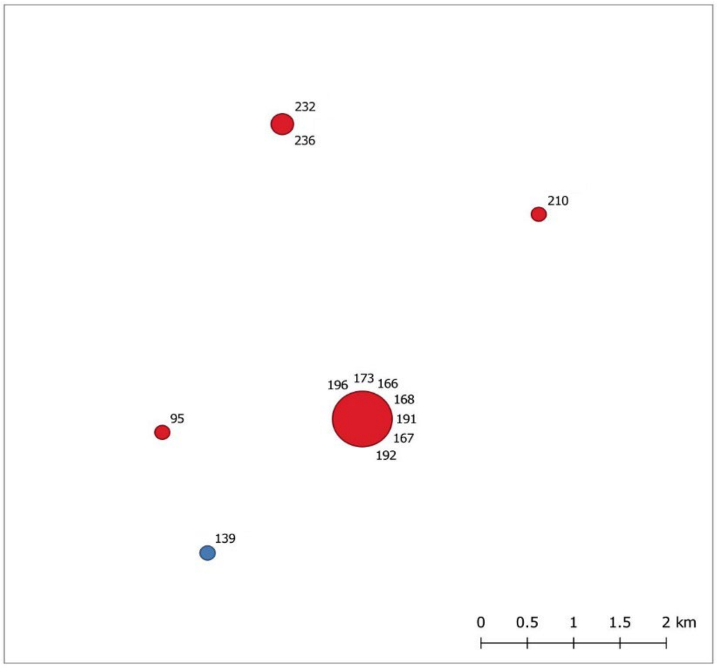

Supplementary Figure S1. The seroconversion map presents the distance and areas in which the participants with definite Zika seroconversion were living in the community. The changes of the participants' serostatus occurred within 6 months are presented in red. One participant with definite seroconversion between the 6- and 12-month visits is highlighted in blue. The map demonstrates a cluster of two symptomatic (ID codes 173 and 191) and five asymptomatic (ID codes 166, 167, 168, 192, 196) infections.
