# Supplementary material for: Seroprevalence of Zika Virus in Amphawa District, Thailand, after the 2016 Pandemic
Source: Viruses. 2022 Feb 25;14(3):476. doi: 10.3390/v14030476 (PMC8953292; doi:10.3390/v14030476)
Supplement: Supplementary file 1 [file viruses-14-00476-s001.zip › viruses-1586007-Table S1.pdf]

**Supplementary Table S1.** Zika virus and dengue virus non-structural protein 1 (NS1) OD of sample/OD of negative control ratio (OD ratio) of 22 Zika seroconverted participants

| ID code | Sero conversion status | History of illness | Laboratory testing | Day 0 | Month 6 | Month 12 |
|---|---|---|---|---|---|---|
| **Participants with Zika NS1 seroconversion between Day 0 and Month 6** | | | | | | |
| 88 | Borderline | Asymptomatic | Zika NS1 | 5.08 | 6.06 | missing |
| | | | Dengue NS1 | 3.10 | 2.87 | missing |
| 178 | Borderline | Asymptomatic | Zika NS1 | 3.17 | 3.15 | 4.11 |
| | | | Dengue NS1 | 1.76 | 1.04 | 1.93 |
| 272 | Borderline | Asymptomatic | Zika NS1 | 10.07 | 11.22 | 12.13 |
| | | | Dengue NS1 | 6.30 | 5.55 | 5.45 |
| 338 | Borderline | Asymptomatic | Zika NS1 | 2.02 | 2.71 | missing |
| | | | Dengue NS1 | 1.29 | 1.31 | missing |
| 95 | Definite | Asymptomatic | Zika NS1 | 1.91 | 12.70 | 5.61 |
| | | | Dengue NS1 | 2.18 | 4.34 | 1.98 |
| 166 | Definite | Asymptomatic | Zika NS1 | 4.12 | 19.67 | 11.96 |
| | | | Dengue NS1 | 3.36 | 6.26 | 3.77 |
| 167 | Definite | Asymptomatic | Zika NS1 | 2.37 | 5.09 | 5.85 |
| | | | Dengue NS1 | 2.90 | 1.69 | 2.34 |
| 168 | Definite | Asymptomatic | Zika NS1 | 1.59 | 14.53 | 4.04 |
| | | | Dengue NS1 | 2.26 | 5.14 | 2.21 |
| 192 | Definite | Asymptomatic | Zika NS1 | 2.16 | 13.50 | 6.97 |
| | | | Dengue NS1 | 2.20 | 6.19 | 2.70 |
| 196 | Definite | Asymptomatic | Zika NS1 | 1.29 | 6.33 | 5.53 |
| | | | Dengue NS1 | 1.38 | 1.97 | 1.40 |
| 232 | Definite | Asymptomatic | Zika NS1 | 2.02 | 15.08 | 6.85 |
| | | | Dengue NS1 | 2.40 | 6.19 | 3.23 |
| 173 | Definite | Symptomatic (Sep 2017, i.e., Month 5; rash without red eyes) | Zika NS1 | 4.62 | 16.59 | 7.11 |
| | | | Dengue NS1 | 4.62 | 5.52 | 3.57 |
| 191 | Definite | Symptomatic | Zika NS1 | 1.38 | 14.93 | 8.99 |
| | | | Dengue NS1 | 1.52 | 5.98 | 2.73 |

| | | | | | | |
|---|---|---|---|---|---|---|
| | | (Sep 2017, i.e., Month 5; rash with red eyes) | | | | |
| 210 | Definite | Symptomatic (Aug 2017, i.e., Month 4; acute febrile illness) | Zika NS1<br>Dengue NS1 | 2.70<br>2.19 | 26.86<br>4.87 | missing<br>missing |
| 236 | Definite | Symptomatic (Jul 2017, i.e., Month 3; acute febrile illness) | Zika NS1<br>Dengue NS1 | 1.72<br>2.16 | 5.19<br>2.08 | 4.02<br>1.76 |
| **Participants with Zika NS1 seroconversion between Month 6 and Month 12** | | | | | | |
| 10 | Borderline | Asymptomatic | Zika NS1<br>Dengue NS1 | 2.94<br>1.39 | 2.85<br>1.53 | 2.90<br>1.37 |
| 55 | Borderline | Asymptomatic | Zika NS1<br>Dengue NS1 | 4.37<br>2.21 | 3.94<br>2.22 | 3.19<br>1.47 |
| 134 | Borderline | Asymptomatic | Zika NS1<br>Dengue NS1 | 4.32<br>2.21 | 4.28<br>2.52 | 5.97<br>1.77 |
| 213 | Borderline | Asymptomatic | Zika NS1<br>Dengue NS1 | 5.12<br>2.57 | 3.44<br>2.15 | 4.30<br>1.66 |
| 228 | Borderline | Asymptomatic | Zika NS1<br>Dengue NS1 | 17.95<br>8.13 | 16.25<br>9.19 | 12.41<br>6.12 |
| 239 | Borderline | Asymptomatic | Zika NS1<br>Dengue NS1 | 10.58<br>8.96 | 8.42<br>4.52 | 8.61<br>4.29 |
| 139 | Definite | Asymptomatic | Zika NS1<br>Dengue NS1 | 1.13<br>1.62 | 1.25<br>1.89 | 3.79<br>1.89 |
